# Human Cytomegalovirus IE2 Disrupts Neural Progenitor Development and Induces Microcephaly in Transgenic Mouse

**DOI:** 10.1007/s12035-023-03310-1

**Published:** 2023-03-29

**Authors:** Delei Niu, Xianjuan Zhang, Shuyun Zhang, Tianyu Fan, Xiaoqiong Zhou, Hui Wang, Xueming Zhang, Fulong Nan, Shasha Jiang, Fengjun Liu, Yunyang Wang, Bin Wang

**Affiliations:** 1 Department of Pathogenic Biology, College of Basic Medicine, Qingdao University, Qingdao, 266000 Shandong China; 2 Department of Immunology, College of Basic Medicine, Qingdao University, Qingdao, 266000 Shandong China; 3 Department of Special Medicine, College of Basic Medicine, Qingdao University, Qingdao, 266000 Shandong China; 4 Department of Endocrinology and Metabolism, the Affiliated Hospital of Qingdao University, Qingdao, 266000 Shandong China

**Keywords:** Human cytomegalovirus, IE2, Neural progenitor cells development, Microcephaly, Transgenic mouse

## Abstract

**Abstract:**

Human cytomegalovirus (HCMV) is a significant contributor to congenital birth defects. Limited by the lack of animal models, the pathogenesis of neurological damage in vivo caused by HCMV infection and the role of individual viral genes remain to be elucidated. Immediate early (IE2) protein may play a function in neurodevelopmental problems caused by HCMV infection. Here, this study intended to investigate IE2’s long-term effects on development of the brain in IE2-expressing transgenic mice (Rosa26-LSL-IE2^+/−^, Camk2α-Cre) aimed to observe the phenotype of postnatal mice. The expression of IE2 in transgenic mice was confirmed by PCR and Western blot technology. We collected mouse brain tissue at 2, 4, 6, 8, and 10 days postpartum to analyze the developmental process of neural stem cells by immunofluorescence. We discovered that transgenic mice (Rosa26-LSL-IE2^+/−^, Camk2α-Cre) can reliably produce IE2 in the brain at various postpartum phases. Furthermore, we also observed the symptoms of microcephaly in postnatal transgenic mice, and IE2 can damage the amount of neural stem cells, prevent them from proliferating and differentiating, and activate microglia and astrocytes, creating an unbalanced environment in the brain’s neurons. In conclusion, we demonstrate that long-term expression of HCMV-IE2 can cause microcephaly through molecular mechanisms affecting the differentiation and development of neural stem cells in vivo. This work establishes a theoretical and experimental foundation for elucidating the molecular mechanism of fetal microcephaly brought by HCMV infection in throughout the period of neural development of pregnancy.

**Graphical Abstract:**

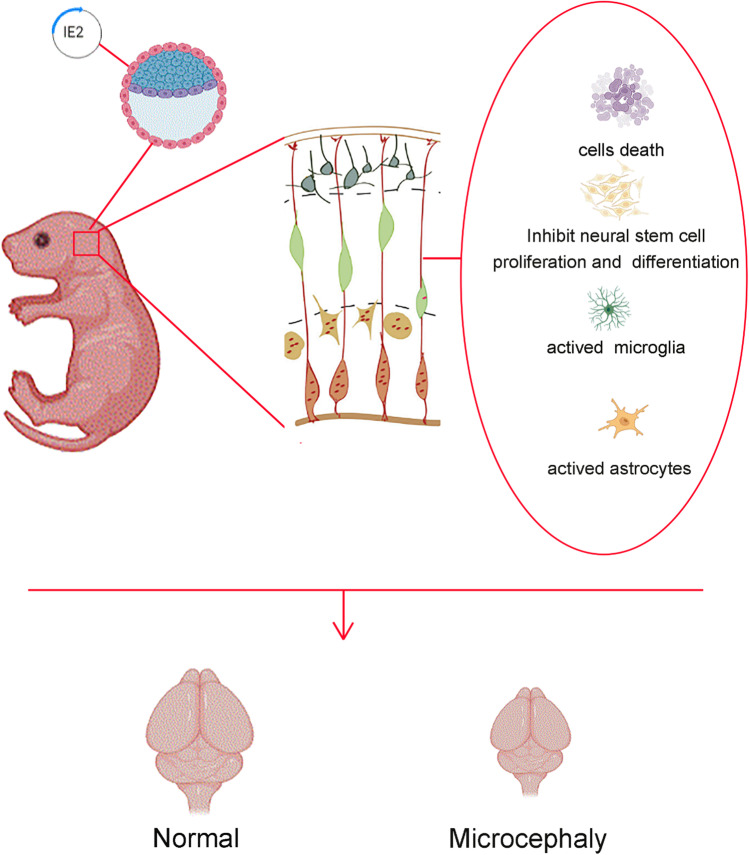

**Supplementary Information:**

The online version contains supplementary material available at 10.1007/s12035-023-03310-1.

## Introduction

Human cytomegalovirus (HCMV), belonging to the *β*-herpesvirus subfamily, is a ubiquitous and adaptable human pathogen that can remain latent for life in infected individuals [1]. HCMV is a kind of opportunistic pathogens, mainly in immunocompromised patients cause significant morbidity and mortality. During pregnancy, HCMV can be transmitted to the developing fetus, thus globally affecting 0.5–2% of newborns [2]. Approximately, 10–15% of neonates with congenital HCMV infection are symptomatic at birth with defects, such as microcephaly (the most serious brain diseases), hearing impairment, mental deficiency, and ventricular calcification [3]. However, the mechanism by which HCMV affects neuropathogenesis has not yet been fully understood.

The correct timing of neural progenitor cell (NPC) proliferation/self-renewal and differentiation, neuronal migration, and maturation is important for appropriate mammalian brain development during normal nervous system development [4–6]. In embryonic stages, NPCs migrate from the ventricular zone (VZ) to the outer surface of the brain to form cortical plate and then differentiate into neurons with different functions as the brain developmental processes progress [7]. However, abnormal proliferation and differentiation of NPCs can lead to neurodevelopmental malformations, such as microcephaly [8, 5, 9]. J. Bradley et al. demonstrated that glia are essential regulators in brain tissue physiology, metabolism, development, and neurological disorders [10–13]. The cerebral cortical glial cells are mainly composed of oligodendrocytes (75.6%), astrocytes (17.3%), and microglia (6.5%) [14]. Late in an embryo’s development or after birth, glial cells form. Glial cells have a large number and complex function in nervous tissues, accounting for 90% of the total number of human central nervous system cells [10, 13]. As a result, appropriate glial cell formation is also required for normal brain volume and function.

An increasing amount of evidence supports that HCMV can infect NPCs and human brain organoids, resulting in dysregulated proliferation and differentiation of NPCs and neuronal death in vitro [15]. Studies of mouse and rhesus monkey CMV in their respective animal models has been used as a route to obtain understanding of HCMV-induced brain malformation in different animal models [16]. Brain organoids were generated from induced pluripotent stem cells (iPSCs) to mimic microcephaly induced in vitro by HCMV infection in three-dimensional human cellular biological systems [17]. However, the pathogenicity, pathogenic mechanism, and pathological damage of different species of CMV are correspondingly very different, and there may be biases in the study of the pathogenic mechanism of HCMV using animal models. Thus, the direct use of animal CMV and its infection model can only provide a limited reference for interpreting the pathogenic mechanism of HCMV, and may not comprehensively resolve the neuropathological problems of HCMV-induced fetal microcephaly. Based on the large and complex genome of HCMV, the specific mechanism of neurological disease caused by HCMV is still unclear. Therefore, there is an urgent need for more research that focuses on examining cellular and molecular alterations at the single-gene level as well as the level of the entire virus.

Immediate-early protein 2 (IE2), encoded by the immediate-early gene *IE2*, is made up of 579 amino acids [18]. As a key transcriptional regulator, IE2 can serve as a standalone host cell promoter and induce the expression of other virus-related genes [19]. In neurological diseases brought on by HCMV infection, IE2 might be crucial. For instance, interference with cell growth regulatory mechanisms by IE2 may be one of the processes by which the virus promotes the development of certain illnesses, such as developmental disorders and microcephaly. Although self-renewal and proliferation of IE2-injured NPCs have also been demonstrated in vitro [20], the effects of IE2 on the growth of NPCs in vivo are unknown at the moment.

Hence, to confirm if IE2 functions can lead to in vivo dysregulation of neurodevelopmental processes, we generated the transgenic mice (Rosa26-LSL-IE2^+/−^, Camk2α-Cre) that can specifically and stably express IE2 in the brain. In this study, we found that IE2 can induce the death of neurons, inhibit the proliferation of NPCs, and activate the activation of microglial cells and astrocytes, ultimately leading to the occurrence of microcephaly. The persistent expression of IE2 in the nervous system results in irreversible damage to the nervous system; thus, this further elucidates better elucidating the neuropathological mechanism of HCMV in causing microcephaly.

## Materials and Methods

### Animals

All animals are kept in an SPF animal house with 12 h of day and night lighting, temperature 23 °C, and water and food supply. Mice are caged at night and seen as vaginal plugs of E0.5 the next day. Animal experiments were carried out in conformity with animal rules and the Welfare and Research Ethics Committee at Qingdao University.

### HCMV-IE2 Conditional Knock-In Mouse Construction

Rosa26-LSL-IE2^+/−^ Mice Are Constructed Using CRISPR/Cas9 Technology

**Fig. 1:**
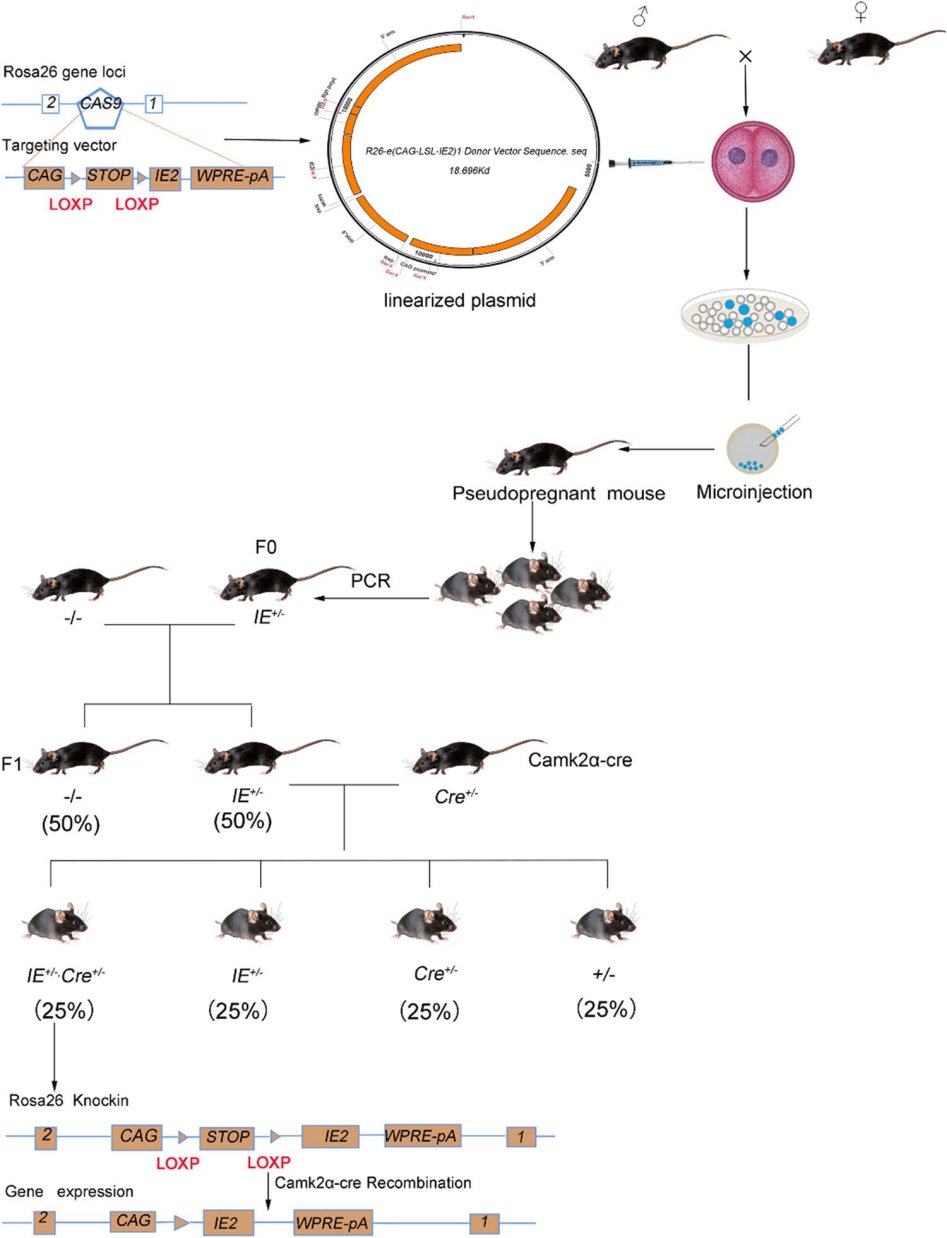
Construction of Rosa26-LSL-IE2^+/−^, Camk2α-Cre mice. Firstly, Rosa26-LSL-IE2^+/−^ transgenic mice were constructed. Next, Rosa26-LSL-IE2^+/−^, Camk2α-Cre mice were generated by crossing Camk2α-Cre mice with Rosa26-LSL-IE2^+/−^ mice. IE2 represents Rosa26-LSL-IE2; Cre represents Rosa26-LSL-IE2, Cre. Amp, ampicillin resistance screening gene; CAG, CAG promoter; loxp, locus of X-overP1; SacII represents SacII restriction endonuclease; STOP represents transcription termination element; WPRE, Woodchuck hepatitis virus posttranscriptional regulatory element. IE2^+/−^ represents Rosa26-LSL-IE2^+/−^; ^−^/^−^ represents wild type; IE2^+/−^, Cre^+/−^ represents Rosa26-LSL-IE2^+/−^, Camk2α-Cre

In brief, using CRISPR, sgRNA was inserted Rosa26 site between exon 1 and exon 2. Cas9 mRNA and gRNA (GGGGACACACTAAGGGAGCTTGG) were obtained by in vitro transcription; a homologous recombination vector (donor vector) was constructed by In-Fusion cloning, which contained a 3.3-kb 5′ homology arm, CAG-LSL-IE2-WPRE-pA, and 3.3-kb 3′ homology arm. The Cas9 mRNA, gRNA, and donor vector were microinjected into the single-cell embryos of C57BL/6J mice to obtain F0 generation mice. The positive F0 generation mice identified by PCR amplification and sequencing were breed with C57BL/6J mice to obtain F1 generation mice (Rosa26-LSL-IE2^+/−^) [21]. Rosa26-LSL-IE2^+/−^, Camk2α-Cre mice were generated by crossing Camk2α-Cre mice with F1 mice (Fig. 1). The enzyme digestion system in Camk2α-Cre mice can remove the STOP component in the hippocampus of Rosa26-LSL-IE2 transgenic mice to achieve conditional overexpression of IE2 protein. The Rosa26-LSL-IE2^+/−^, Camk2α-Cre mice were identified by PCR. The Rosa26-LSL-IE2^+/−^ mice were control mice. Rosa26-LSL-IE2^+/−^, Camk2α-Cre mice were experimental group. The sequences of primers were listed in Table 1 [22].

**Table 1:** The sequence of real-time PCR primers of Rosa26-LSL-IE2^+/−^, Camk2α-Cre mice

Primers name	Forward (5′-3′)	Reverse (5′-3′)	Product size
Wild type	TCAGATTTTTATAGGGGACACA	TAAAGGCCACTCAATGCTCACTAA	994 bp
IE2	TCAGATTCTTTATAGGGGACACA	ACGCTATGTGGATACGCTGCT	414 bp
Camk2α-Cre	GGGGAGGTAGGAAGAGCGATGA	CATCGACCGGTAATGCAG	800 bp

### Sample Collection

The brain tissues of P2, P4, P6, P8, and P10 mice were taken out and fixed in 4% paraformaldehyde for 24 h. And to embed the brain tissue in paraffin, cut 2–3 μm brain section.

### DNA Extraction and PCR

The mice were separated into two groups: experimental mice and control mice, cut off in the 0.2-cm mouse tail, extracted DNA according to the kit provided by Novizan, and identified by PCR. The experimental reaction steps are as follows: pre-denaturation at 94 °C for 5 min, followed by 35 cycles at 94 °C for 30 s; annealing at 55 °C for 35 s; extension at 72 °C for 1 min; at 72 °C extend 10 min further.

### HE Staining

Paraffin sections were dewaxed, then hydrated for further H&E staining, dehydrated, and then transparent for microscopic observation.

### Nissl Stain

After routine dehydration of paraffin sections, Brain sections were stained for 15 min with 0.1% toluidine blue before being dehydrated for 2 min in 70%, 80%, and 95% ethanol. Finally, the sections were transparentized with xylene for more than 30 min and observed under an optical microscope (from Olympus, Japan).

### Immunofluorescence

Paraffin sections were deparaffinized/hydrated, placed in water for 5 min, and then subjected to antigen retrieval in a pressure cooker using 0.01 M pH 9.0 Tris-EDTA retrieval solution for 10 min. Sections were blocked in 5% bovine serum albumin (BSA) and 0.05% Triton X-100 for 30 min, and the primary antibody was added dropwise and incubated overnight at 4 °C. The next day, sections were washed, blocked for an additional 15 min, and incubated with secondary antibody at 1:500 for 60 min at room temperature. Sections were washed, incubated with DAPI containing fluorescent inhibitor for 5 min, and then sealed. Primary antibodies are as follows: IBA-1(1:100, ABclonal, cat. no. A19776), CD68 (1:100, ABclonal, cat. no. A6554), MBP (1:100, ABclonal, cat. no. A11162), CNP (1:100, ABclonal, cat. no. A19033), GFAP (1:100, ABclonal, cat. no. A119058), PAX6 (1:100, ABclonal, cat. no. A19099), Ki67 (1:100, ABclonal, cat. no. A16919), TBR1 (1:100, ABclonal, cat. no. A19550), SOX2 (1:100, ABclonal, cat. no. A0561), S100 *β* (1:100, BOSTER, cat. no. BM4087), NEUN (1:100, BOSTER, cat. no. BM4354), Caspase 3 (1:100, affinity, cat. no. BF0711), Camk2α (1:100, Abbkine, cat. no. ABP56642), CMV-IE1 and IE2 (1:100, Abcam, cat. no. ab53495), and Ki67 (1:100, Servicebio, mouse, GB121141). Secondary antibodies (1:500) were used as follows: goat anti-mouse-Cy3 (Bioss) and goat anti-rabbit FITC (Bioss). Images were taken using an OLYMPUS FLUOVIEW FV1200 microscope and processed with ImageJ software, as specified in the figure legends.

### Western Blot

The cerebral cortex and hippocampus were isolated and triturated and subjected to radioimmunoprecipitation assay (RIPA) buffer. An equivalent amount of cell lysate (an important component) was separated by sodium dodecyl sulfate polyacrylamide gel electrophoresis (SDS-PAGE) and transferred to a PVDF membrane (Millipore). After incubation with the primary antibody overnight at 4 °C, the corresponding secondary antibody was incubated at room temperature for 2 h, and electrochemiluminescence (ECL) was dropped for 1 min. The signal was detected using a chemiluminescence instrument (ImageQuant LAS 500, Sweden). Primary antibodies are as follows: CMV-IE1 and IE2 (1:500, Abcam, cat. no. ab53495) and *β*-actin (1:2000, Santa Cruz). Secondary antibodies used were horseradish peroxidase conjugated sheep anti-mouse IgG (1:500, Absin) and anti-rabbit IgG (1:500, Absin) (1:10,000, Absin).

### Confocal Imaging and Quantification

Brain slices were scanned on an OLYMPUS FLUOVIEW FV1200 microscope and processed with Imaris, ImageJ, and Photoshop, as previously described [23].

### Statistical Analysis

All data were analyzed using GraphPad Prism V8.0 (GraphPad Software, Inc., La Jolla, CA, USA). The data were expressed as mean ± SEM and analyzed by two-tailed Wilcoxon rank-sum test, *P < 0.05, **P < 0.01, ***P < 0.001, ****P < 0.0001.

## Results

### HCMV-IE2 Is Specifically Expressed in the Brain and Causes Microcephaly

**Fig. 2:**
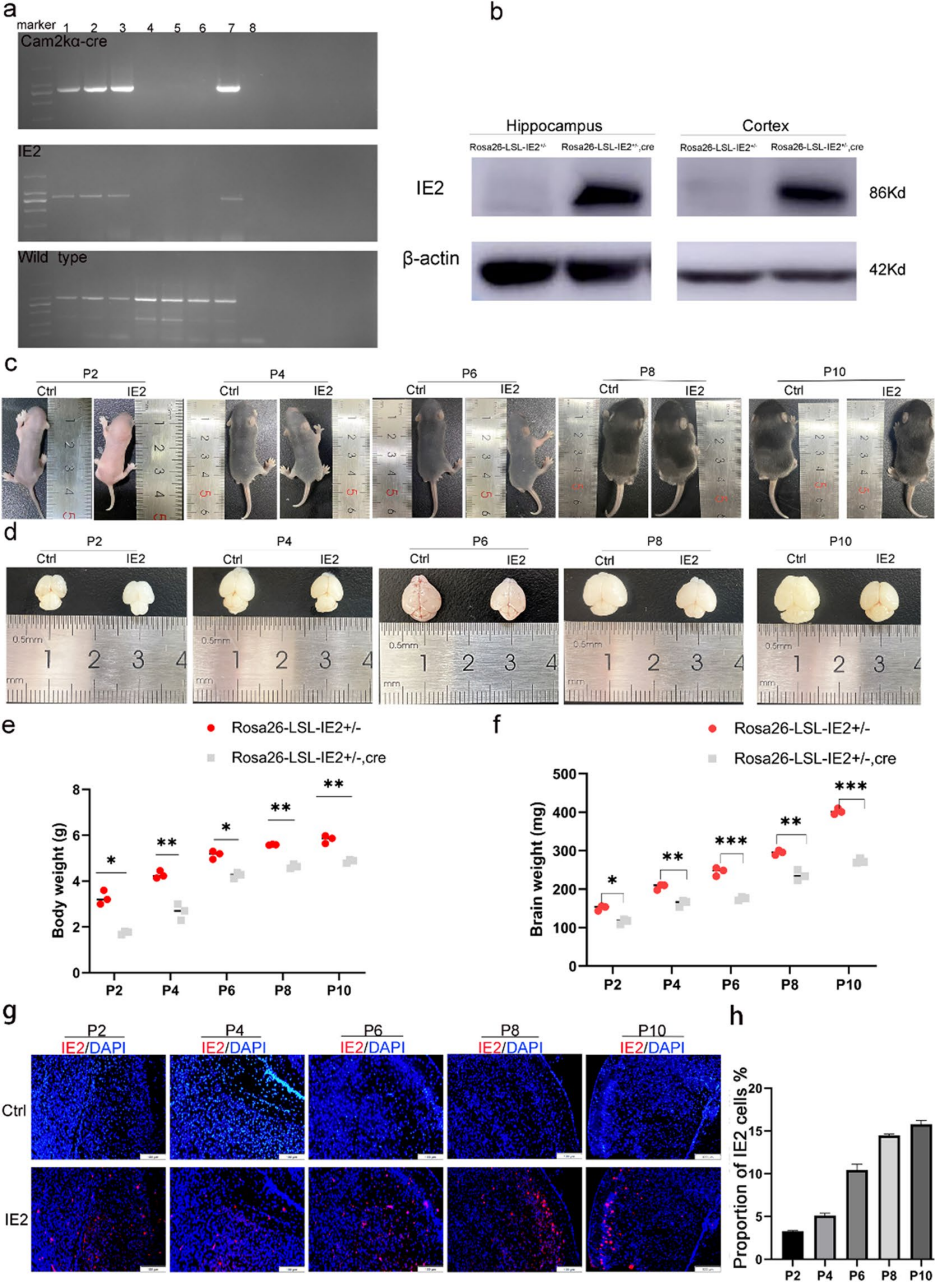
HCMV-IE2 is specifically expressed in the brain and causes microcephaly. **a** PCR identification of Rosa26-LSL-IE2^+/−^, Camk2α-Cre mice (lanes 1–3) Rosa26-LSL-IE2^+/−^, Camk2α-Cre mice, (lanes 4–6) wild-type mice, (lane 7) the positive mice, (lanes 8) the H_2_O control. PCR product size is as follows: IE2 819 bp, Camk2α-Cre 800bp, wild-type 994bp. **b** Western blotting verified the expression of IE2 in hippocampus and cortex of Rosa26-LSL-IE2^+/−^, Camk2α-Cre mice. **c** Morphology of whole body at stages P2, P4, P6, P8, and P10. *n* = 3, the number of P2, P4, P6, P8, P10 brain. **d** Morphology of brain at stages P2, P4, P6, P8, and P10. *n* = 3, the number of P2, P4, P6, P8, P10 brain. **e** Weight of body at stages P2, P4, P6, P8, and P10. **f** Weight of brain at stages P2, P4, P6, P8, and P10. **g** The expression of IE2 in the cerebral cortex at stages P2, P4, P6, P8, and P10 was detected by immunofluorescence staining. Scale bar, 100 μm. P2, *n* = 9/3; P4, *n* = 9/3; P6, *n* = 9/3; P8, *n* = 9/3; P10, *n* = 9/3. *n*, number of slices/different brains. **h** Statistical chart of the proportion of IE2 expression in the cerebral cortex of Rosa26-LSL-IE2^+/−^, Camk2α-Cre mice. Ctrl represents Rosa26-LSL-IE2^+/−^ mice; IE2 represents Rosa26-LSL-IE2^+/−^, Camk2α-Cre mice. Error bars indicate SEM of nine sections from three independent experiments (*P < 0.05, **P < 0.01, ***P < 0.001, ****P < 0.0001)

To explore the role of IE2 in brain development, we used CRISPR/Cas9 technology to construct IE2-expressing mice (Rosa26-LSL-IE2^+/−^, Camk2α-Cre), which can stably and continuously express HCMV-IE2 in the brain. Genotyping of mice expressing IE2 was performed by polymerase chain reaction (PCR) (Fig. 2a) and further confirmed by Western blot (Fig. 2b). Camk2α and IE2 co-staining results also prove the ectopic expression of IE2 in camk2a-positive cells (Supplementary Fig. 1). The results showed that IE2 protein can be specifically expressed in both cerebral cortex and hippocampus.

Subsequently, we measured the overall morphology of body size and brain of the IE2-expressing mice during the postnatal 2 to 10 days. Interestingly, we found that IE2-expressing mice exhibited smaller body size and brain morphology than control mice of the same age from stage P2 through P10 (Fig. 2c, d). Additionally, the body weight and brain weight of the IE2-expressing mice were significantly lower than the control mice of the same age (Fig. 2e and f). However, there was no difference in size and weight in the other organs (heart, lung, liver, and kidney) of IE2-expressing mice compared with the control group (Supplementary Fig. 2a and b).

Furthermore, to evaluate IE2 expression in the brain, we examined brain tissues obtained at stages P2, P4, P6, P8, and P10 by immunofluorescence (Fig. 2g). The results showed that the IE2 is mainly expressed in the cortical lamellar, ependymal area, and subependymal area along with the hippocampus. As illustrated in Fig. 2h, the IE2 expression level gradually increased in the cerebral cortex and hippocampus with the brain development. According to the data, HCMV-IE2 causes significant developmental problems in the nervous system and causes microcephaly during the active period of neurodevelopmental processes.

### HCMV-IE2 Causes Thinning of the Cerebral Cortex

**Fig. 3:**
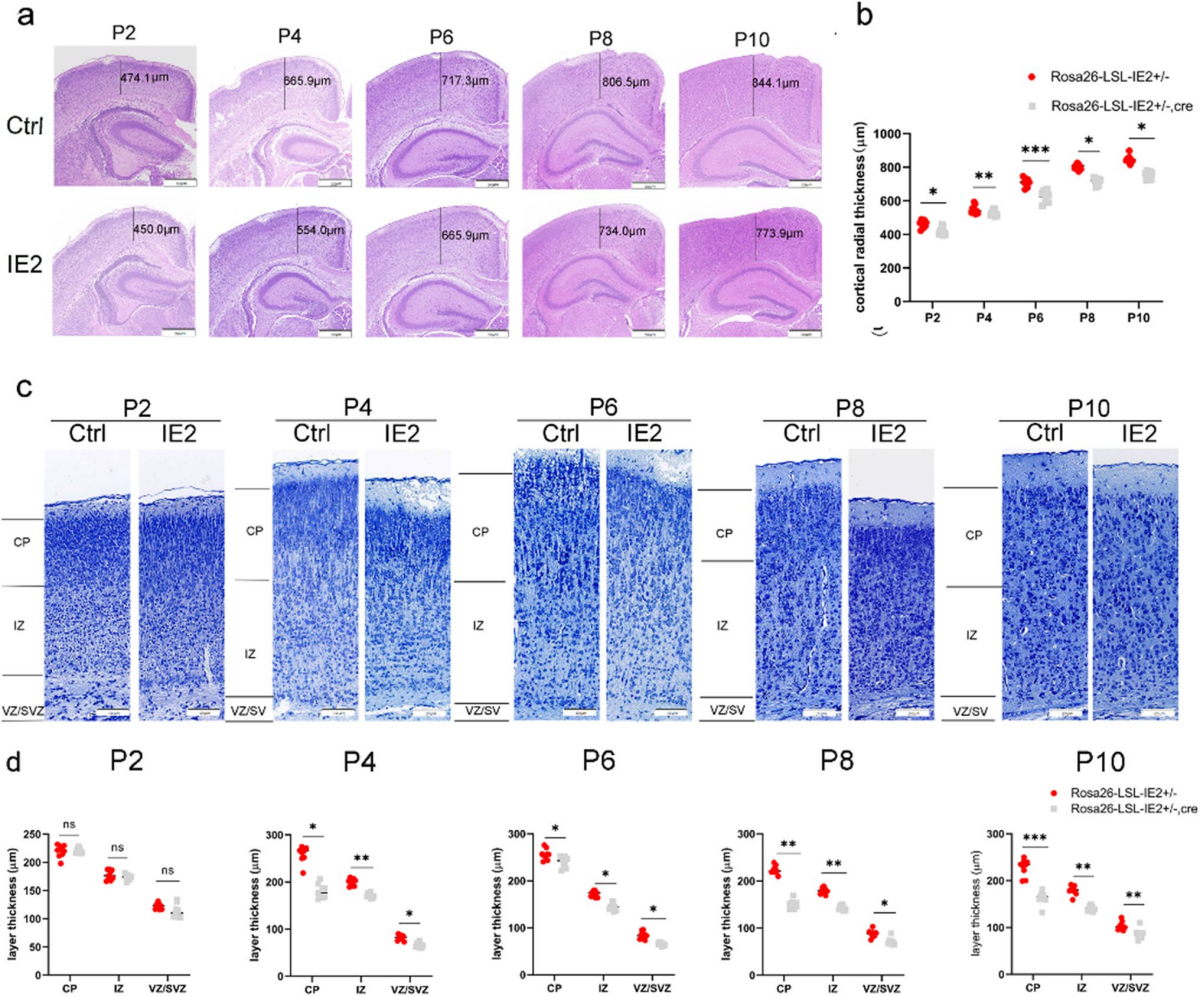
HCMV-IE2 causes cortical thinning and hippocampal area reduction. **a** Hematoxylin-eosin (H&E) staining was performed on coronal slices of the cerebral cortex at phases P2, P4, P6, P8, and P10. Scale bars, 200 μm. The numbers in the figure indicate the thickness of the cortical brain. **b** Statistical analysis of radial thickness of cerebral cortex from the experiment shown in **a**. **c** Hippocampus area at stages P2, P4, P6, P8, and P10 stained with Nissl staining. Scale bar, 200 μm. **d** Quantification of hippocampus area from the experiment shown in **c**. Ctrl represents Rosa26-LSL-IE2^+/−^ mice; IE2 represents Rosa26-LSL-IE2^+/−^, Camk2α-Cre mice. Error bars indicate SEM of nine sections from three independent experiments (ns represents no statistical difference, *P < 0.05, **P < 0.01, ***P < 0.001, ****P < 0.0001). P2, *n* = 9/3; P4, *n* = 9/3; P6, *n* = 9/3; P8, *n* = 9/3; P10, *n* = 9/3. *n*, number of slices/different brains

To understand the potential mechanism of microcephaly associated with IE2, we subsequently focused on brain development. As expected, the radial thickness of the cerebral cortex was significantly reduced in Rosa26-LSL-IE2^+/−^, Camk2α-Cre mice compared with control mice at the stages P2, P4, P6, P8, and P10 (Fig. 3a and b).

Neurons travel from proliferative areas to the cortex’s outer membrane region and develop to establish an orderly hierarchical structure [24]. This process is precisely regulated. Once affected by external factors, it must correspondingly affect the brain structure. To determine whether the normal structures of the cortical layers in the IE2-expressing mice were disrupted, and to delineate which layers of neurons were affected, we carried out Nissl staining on the cerebral cortex. As shown in Fig. 3c and d, the overall organizational structures of the cerebral cortical layers in the IE2-expressing mice were similar to those of the control mice. However, the thickness of the ependymal zone/subependymal zone (VZ/SVZ), intermediate zone (IZ), and cortical plate zone (CP) of the cerebral cortex of IE2-expressing mice was thinner than that of the control mice at the stages P4, P6, P8, and P10.

### HCMV-IE2 Causes Neuronal Loss Which Leads to Neuronal Cell Death

**Fig. 4:**
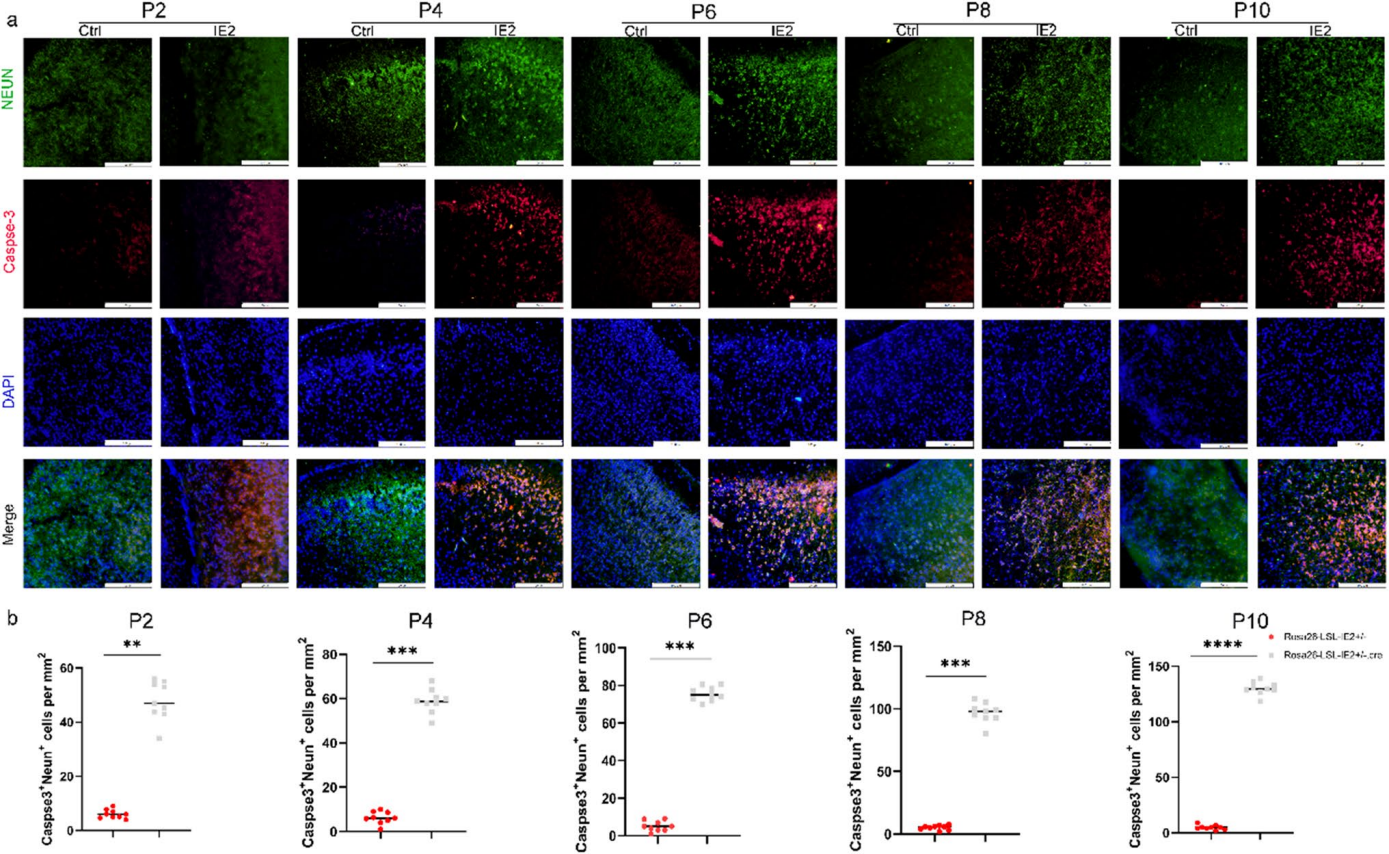
HCMV-IE2 causes neuronal loss through neuronal cell death. **a** Images of the cerebral cortex was co-stained with Caspase-3 and NEUN at stages P2, P4, P6, P8, and P10. Caspase-3 (red). NEUN (greed). DAPI (blue). Scale bar, 50 μm. **b** Statistical analysis of Caspase-3 NEUN cells per mm^+^^+^^2^ at stages P2, P4, P6, P8, and P10 cerebral cortex from the experiment shown in **a**. Ctrl represents Rosa26-LSL-IE2^+/−^ mice; IE2 represents Rosa26-LSL-IE2^+/−^, Camk2α-Cre mice. Error bars indicate SEM of nine sections from three independent experiments (*P < 0.05, **P < 0.01, ***P < 0.001, ****P < 0.0001). P2, *n* = 9/3; P4, *n* = 9/3; P6, *n* = 9/3; P8, *n* = 9/3; P10, *n* = 9/3. *n*, number of slices/different brains

To investigate the reason of cerebral cortex thinning, we used NEUN immunofluorescence labelling to count the total number of neurons in the brain. The results showed that compared to control group, the total number of neurons in as IE2-expressing brains was drastically reduced from P2 to P10 (Fig. 4a).

Microcephaly has been linked to increased cell death [25], and to determine whether IE2 expression induces the apoptosis of nerve cells in the brain, we co-stained with the marker Caspase-3 of apoptosis and the marker NEUN of neurons; to see, observe apoptosis of neurons after birth (Fig. 4a and b). Compared to the control group, at P2 days, a large number of apoptosis signals can be detected in the cortical laminar area of the brain of Rosa26-LSL-IE2^+/−^, Camk2α-Cre mice. Our results showed that with the development of the brain, long-term expression of IE2 significantly enhances the apoptosis of nerve cells in the brain, leading to the thinning of the cerebral cortex.

### HCMV-IE2 Leads to Depletion of Cortical Neural Progenitors

**Fig. 5:**
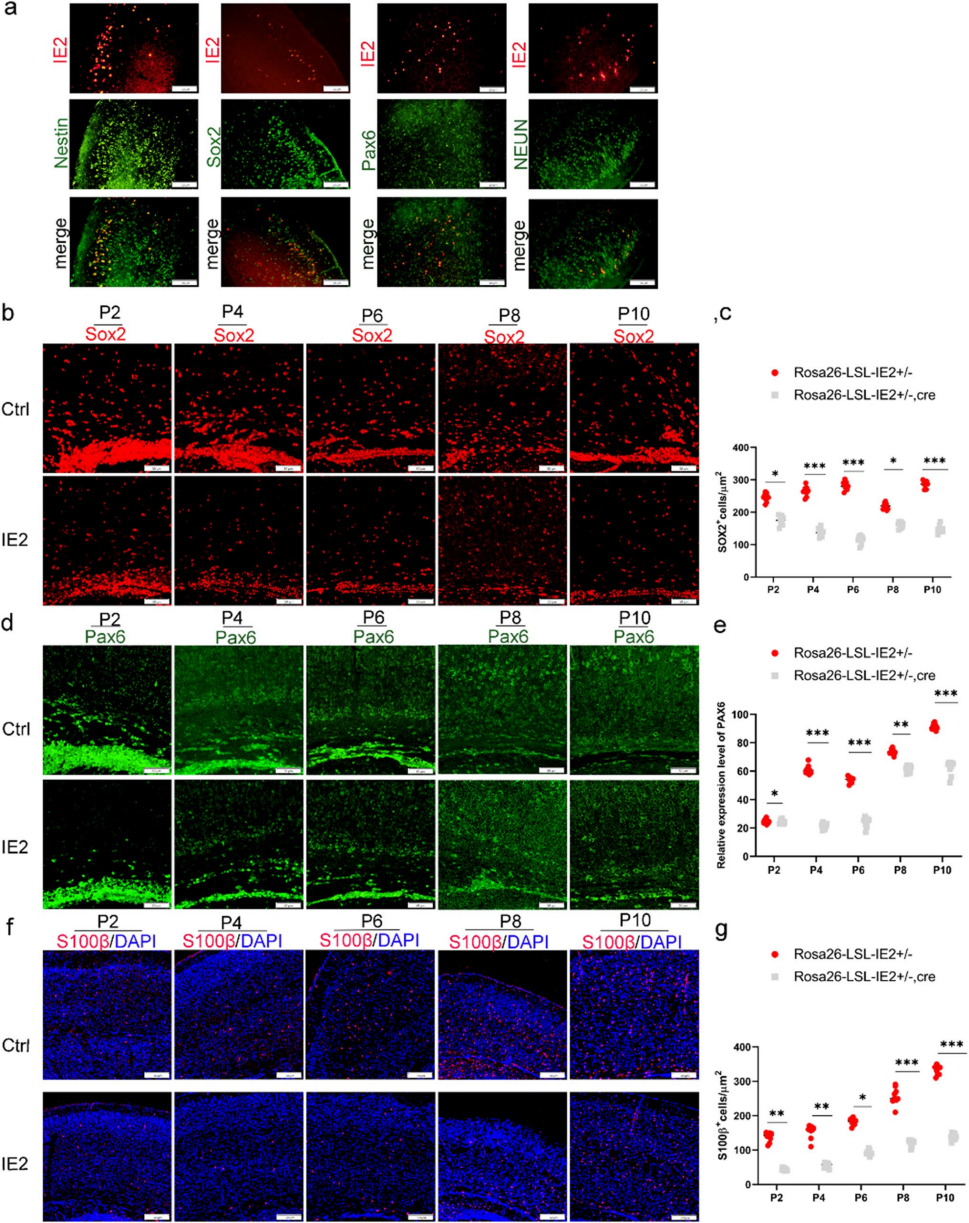
HCMV-IE2 leads to depletion of cortical neural progenitors. **a** Immunofluorescence IE2 and neuronal markers co-stained from left to right as IE2 (red) and NESTIN (greed), IE2 and SOX2 (greed), IE2 and PAX6 (greed), IE2 and NEUN (greed). Scale bar, 100 μm. **b** Immunofluorescence for SOX2 showed APCs at stages P2, P4, P6, P8, and P10 of cerebral cortex. SOX2 (red) DAPI (blue). Scale bars, 50 μm. **c** Quantification of SOX2 cells per μm^+^^2^ at stages P2, P4, P6, P8, and P10 cerebral cortex from the experiment shown in **b**. **d** Immunofluorescence for PAX6 showed RGCs at stages P2, P4, P6, P8, and P10 of cerebral cortex. PAX6 (green) DAPI (blue). Scale bars, 50 μm. **e** Quantification of relative repression level of PAX6 at stages P2, P4, P6, P8, and P10 cerebral cortex from the experiment shown in **d**. **f** Immunofluorescence for S100β showed OPCs at stages P2, P4, P6, P8, and P10 of cerebral cortex. S100β (red) DAPI (blue). Scale bars, 100 μm. **g** Quantification of S100β cells per μm^+^^2^ at stages P2, P4, P6, P8, and P10 cerebral cortex from the experiment shown in **f**. Ctrl represents Rosa26-LSL-IE2^+/−^ mice; IE2 represents Rosa26-LSL-IE2^+/−^, Camk2α-Cre mice. Error bars indicate SEM of nine sections from three independent experiments (*P < 0.05, **P < 0.01, ***P < 0.001, ****P < 0.0001). P2, *n* = 9/3; P4, *n* = 9/3; P6, *n* = 9/3; P8, *n* = 9/3; P10, *n* = 9/3. *n*, number of slices/different brains

Subsequently, we assessed whether the reduced number of neurons in the cortex of IE2-expressing mice could be due to fewer numbers of NPCs. First, to determine which cell types express IE2 in the transgenic mouse brain, we examined P10 brain sections with cell markers including Nestin, an NSC marker; Sox2, an apical progenitor cell marker; Pax6, a radial glial cell marker; and NEUN, a mature neuron marker. As shown in Fig. 5a, IE2-expressing cells express Nestin, SOX2, PAX6, and NEUN, indicating that IE2 is mostly expressed in NPCs and neurons.

Next, to investigate the effect of IE2 on the amount of NPCs, we performed the SOX2 immunofluorescence staining on brain sections at the stages P2, P4, P6, P8, and P10. From P2 to P10, the amount of apical precursor cells in IE2-expressing mice was considerably lower than in control mice (Fig. 5b and c). As the brain developmental processes progress, the apical precursor cells further develop into a new type of neural precursor cells, namely radial glial cells. Surprisingly, we discovered that the amount of PAX6-positive cells was significantly reduced in IE2-expressing mice starting at stage P2 (Fig. 5d and e), indicating that radial glial cells are also significantly blocked at critical moments of neural development, affecting the development of the entire NPCs. Furthermore, we also analyzed the glial progenitor cells using S100β as a marker of astrocytes and oligodendrocyte progenitor cells. The fewer S100β-positive cells were detected in the cerebral cortex of IE2-expressing mice at stages P2, P4, P6, P8, and P10 (Fig. 5f and g). All of these findings suggested that HCMV-IE2 causes a decrease in the number of neural stem cells, which could also be the cause of cerebral cortex thinning.

### HCMV-IE2 Influences Neural Stem Cell Proliferation and Differentiation

**Fig. 6:**
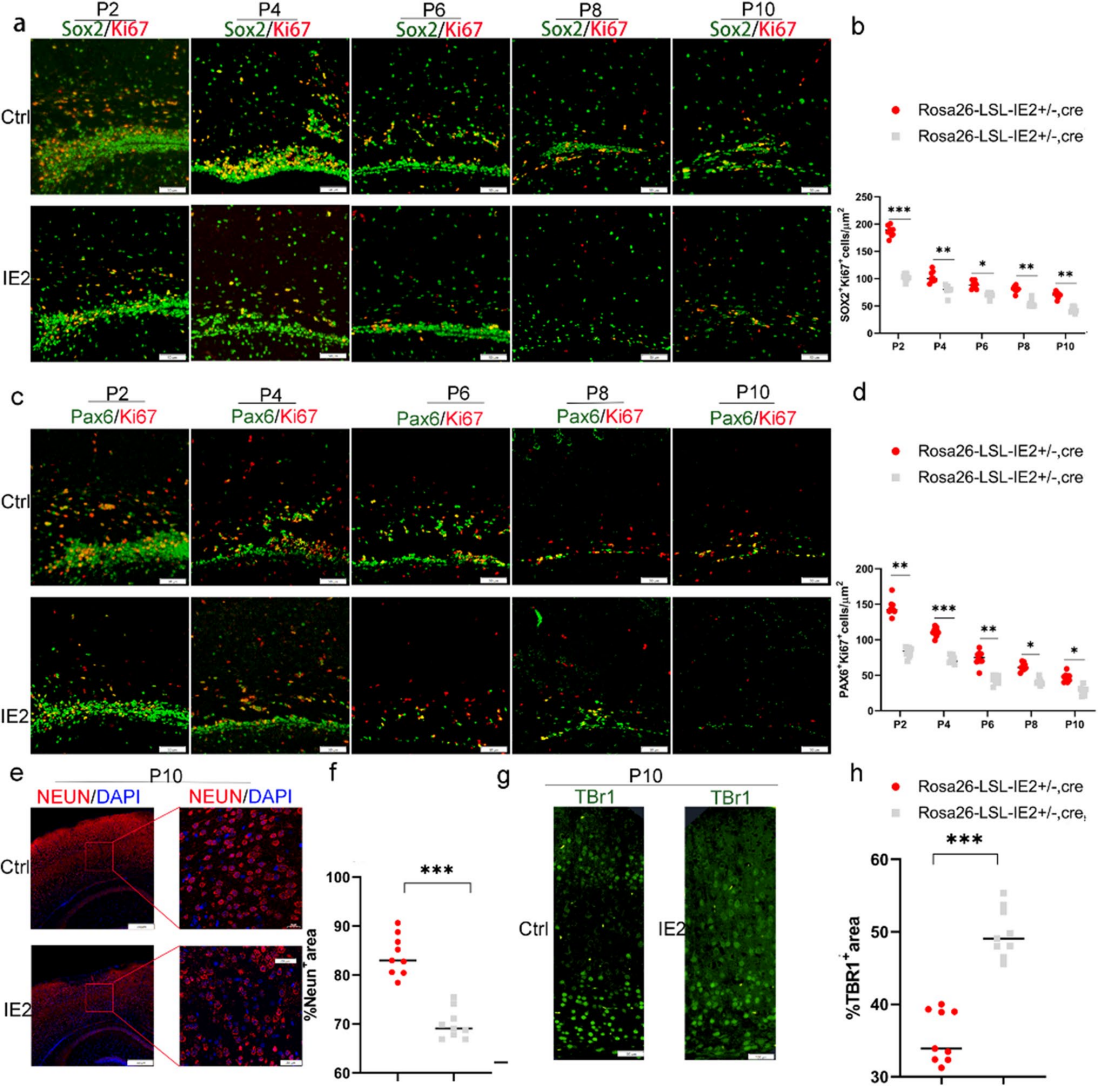
HCMV-IE2 affects the proliferation and differentiation of neural stem cells. **a** Immunofluorescence for P2, P4, P6, P8, and P10 cerebral cortex, with primary antibodies against SOX2 (greed), Ki67 (red), and Alexa Fluor 488- and 555-conjugated secondary antibodies. Scale bar, 50 μm. **b** Ki67SOX2 cells per μm^+^^+^^2^ at stages P2, P4, P6, P8, and P10 cerebral cortex from the experiment. **c** Immunofluorescence for P2, P4, P6, P8, and P10 cerebral cortex, with primary antibodies against Ki67 (red), PAX6 (greed), and FITC- and CY3-conjugated secondary antibodies. Scale bar, 50 μm. **d** Statistical analysis of Ki67PAX6 cells per μm^+^^+^^2^ at stages P2, P4, P6, P8, and P10 cerebral cortex from the experiment shown in **c**. **e** P10 cerebral cortex stained with immunostained for NEUN (red). DAPI (blue). The territories in the **e** red boxes are expanded in the right panels. Scale bar, 100 μm (**f**); 50 μm (**h**). **f** Statistical analysis of %TBR1 area at stage P10 cerebral cortex from the experiment presented in **e**. **g** P10 cerebral cortex stained with immunostained for TBR1 (green). DAPI (blue). Scale bar, 100 μm. **h** Quantification of %TBR1 area at stage P10 cerebral cortex from the experiment shown in **g**. Ctrl represents Rosa26-LSL-IE2^+^^+^^+/−^ mice; IE2 represents Rosa26-LSL-IE2^+/−^, Camk2α-Cre mice. Error bars indicate SEM of nine sections from three independent experiments (*P < 0.05, ** *P* < 0.01, ***P < 0.001, ****P < 0.0001). P2, *n* = 9/3; P4, *n* = 9/3; P6, *n* = 9/3; P8, *n* = 9/3; P10, *n* = 9/3. *n*, number of slices/different brains

The specific expression of Camk2α in neural stem cells (NSCs) and neurons [1] led to the activation of IE2 transcription and translation processes in both cells of our mouse model. Based on the lower number of neurons in the cerebral cortex of IE2-expressing mice, we suspected that HCMV-IE2 might disrupt the proliferation of NSCs. To confirm this possibility, Ki67 immunofluorescence was used to examine the proliferation ability of neural stem cells. Ki67 is a nuclear antigen found in proliferating cells. As shown in Fig. 6a and b, the number of Ki67SOX2 cells was significantly lower in IE2-expressing mice than that in the control mice from P2, suggesting that HCMV-IE2 affects the proliferation of apical precursor cells in brain tissue. Additionally, the number of PAX6Ki67 cells is also significantly decreased in IE2-expressing mice (Fig. ^+^^+^^+^^+^6c and d), indicating that radial glial cell proliferation is also inhibited.

Next, we examined whether IE2 affects the differentiation of neural stem cells. TBR1 is a marker for immature neurons [26], while NEUN is a marker for mature neurons [26–28]. Compared with the control group, TBR1-positive cells were increased, and NEUN-positive cells were remarkably decreased in the cerebral cortex of IE2-expressing mice at P10 stages (Fig. 6e–h). As a result, our findings suggest that HCMV-IE2 may impede neural stem cell proliferation and delay neural stem cell differentiation, resulting in severe microcephaly, but it is subject to evaluation in further studies.

### HCMV-IE2 Causes the Activation of Microglia

**Fig. 7:**
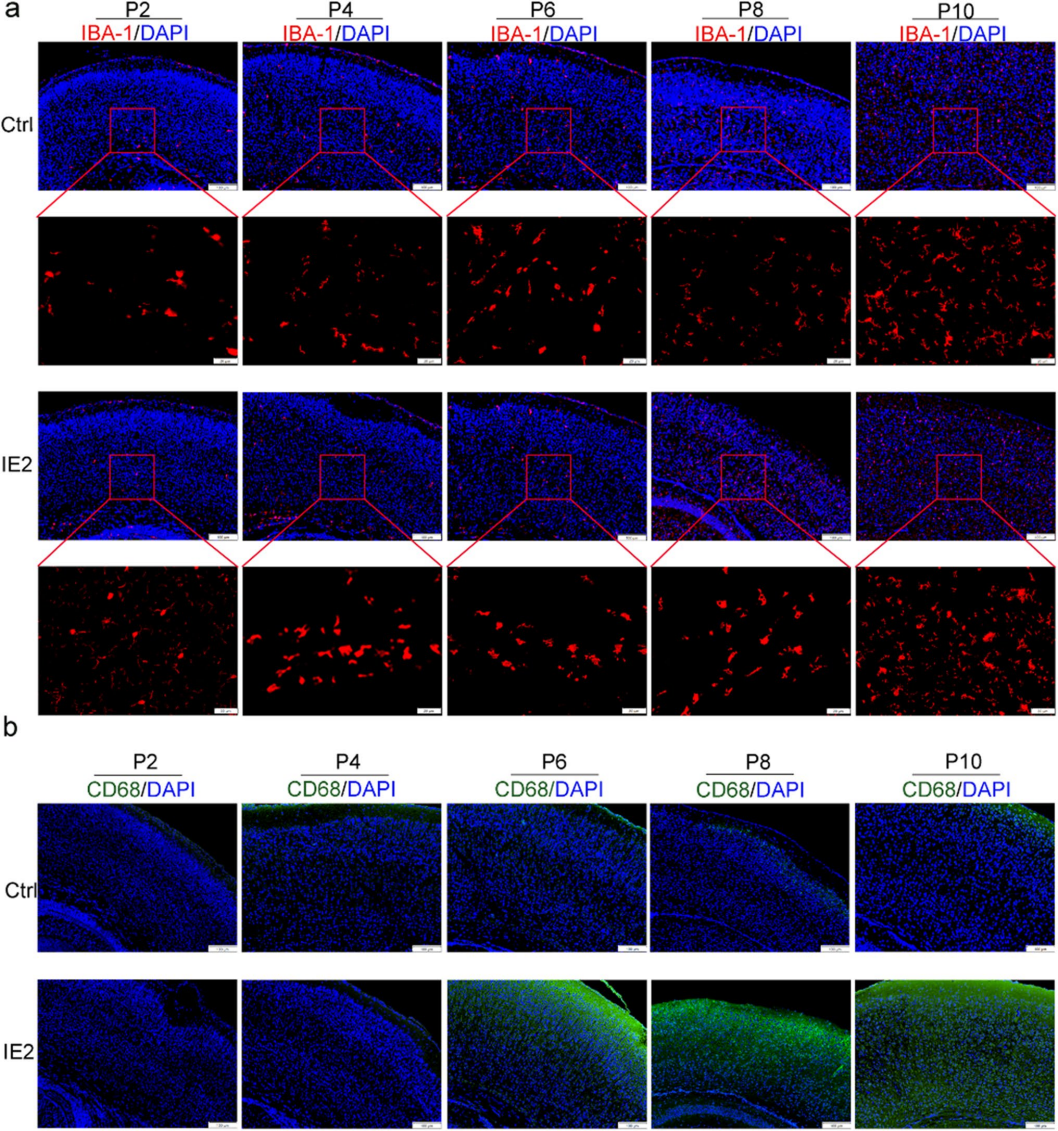
HCMV-IE2 causes the activation of microglia. **a** Immunofluorescence staining of brain coronal sections using IBA-1 (red) at stages P2, P4, P6, P8, and P10. Scale bars, 100 μm and 20 μm. **b** Immunofluorescence staining of brain coronal sections using CD68 (greed), DAPI (blue) at stages P2, P4, P6, P8, and P10. Scale bar, 100 μm. Ctrl represents Rosa26-LSL-IE2^+/−^ mice; IE2 represents Rosa26-LSL-IE2^+/−^, Camk2α-Cre mice. P2, *n* = 9/3; P4, *n* = 9/3; P6, *n* = 9/3; P8, *n* = 9/3; P10, *n* = 9/3. *n*, number of slices/different brains

Microglia, central nervous system innate immune cells, can also transition into an active state and perform their functions for defend against abnormalities of the nervous system abnormalities [4, 10, 29]. In the activated state, the plasma cells of the microglia changed from a branching shape to round and increased in number. We stained the cortices with IBA-1 to determine how IE2 affects microglial cells (a marker for microglia). At stages P2 and P4, weak signals of IBA-1 were observed, while at stages P6, P8, and P10, strong signals of IBA-1 were detected in the cerebral cortex of IE2-expressing mice (Fig. 7a each group above). Furthermore, compared with the control mice, the morphology of microglia also changed from branched shape to round in the cerebral cortex of IE2-expressing mice, indicating that IE2 activates the function of microglia (Fig. 7a each group below). The majority of IBA-1 cells were also positive for CD68, a phagolysosome and active microglia marker, indicating the clear activation of microglia. As shown in Fig. ^+^7b and Supplementary Fig. 3, the CD68-positive signals were also strongly expressed in the cerebral cortex of IE2-expressing mice from stage P6, which further confirmed that IE2 can activate microglia. Our data indicated that IE2 may induce inflammation in the brain tissues and cause persistent activation of microglia, resulting in brain tissue damage.

### HCMV-IE2 Induces the Transformation of Protoplasmic Astrocytes into Reactive Astrocytes

**Fig. 8:**
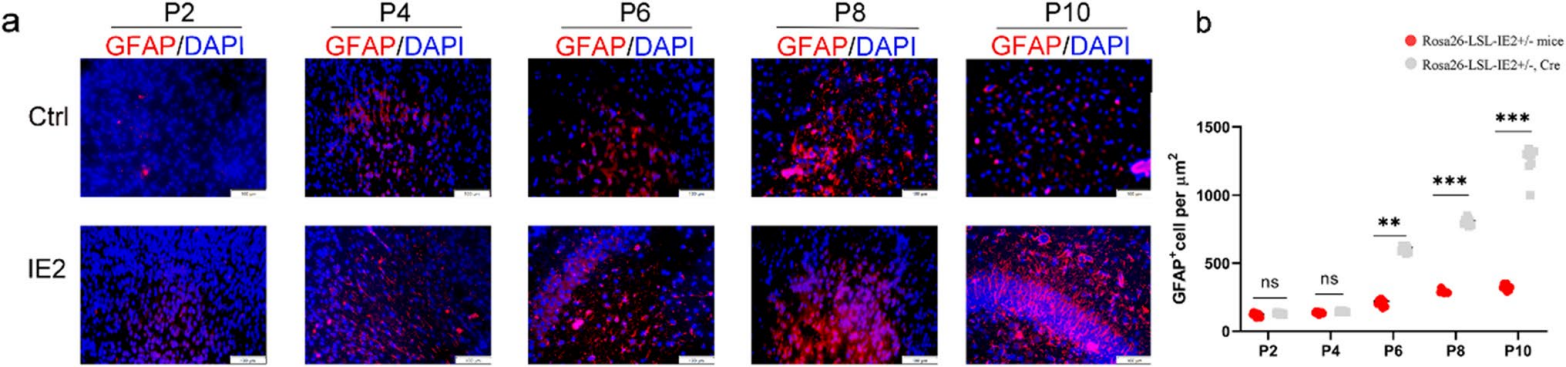
HCMV-IE2 induces the transformation of protoplasmic astrocytes into reactive astrocytes. **a** Immunofluorescence staining of brain coronal sections using GFAP at stages P2, P4, P6, P8, and P10. GFAP (red). DAPI (blue). Scale bar, 100 μm. **b** Measurement of GFAP cells per μm^+^^2^ at stages P2, P4, P6, P8, and P10 cerebral cortex from the experiment presented in **a**. Ctrl represents Rosa26-LSL-IE2^+/−^ mice; IE2 represents Rosa26-LSL-IE2^+/−^, Camk2α-Cre mice. Error bars indicate SEM of nine sections from three independent experiments (ns, no statistical difference; *P < 0.05, **P < 0.01, ***P < 0.001). P2, *n* = 9/3; P4, *n* = 9/3; P6, *n* = 9/3; P8, *n* = 9/3; P10, *n* = 9/3. *n*, number of slices/different brains

Astrocytes are the most abundant type of cell in the central nervous system, giving nutrition, managing the chemical environment, and scavenging neurotransmitters for neuronal cells [14]. To explore whether HCMV-IE2 affects astrocytes, we checked the astrocyte population and morphology by immunofluorescence with anti-GFAP (a marker for astrocytes) antibodies. The results revealed that there was no change in GFAP expression between the two different groups at stage P2. While compared with control mice, significantly increased GFAP-positive signals were detected in the IE2-expressing mice and increased continuously from stage P4 through P10 (Fig. 8a). Notably, In IE2-expressing animals, GFAP-positive cells displayed an active glial morphology, indicating that astrocytes were gradually stimulated as well (GFAP is exclusively found in white matter glia and fibrillar astrocytes in healthy brains). Strong GFAP expression and morphological changes in the cortex indicate the conversion of protoplasmic astrocytes to their reactive counterparts (Fig. 8b). Taken together, these findings suggest that HCMV-IE2 may cause progressive microglial activation and astrogliosis, lending credence to the concept that HCMV-IE2 causes brain damage.

## Discussion

HCMV can be transmitted vertically through mother to infant, and it is an important pathogen involved in teratogenic and neurological damage pathogen in humans. The damage wrecked by HCMV infection to the fetus during pregnancy is mainly connected with the central nervous system. Damage to any of the major cells that make up the nervous system (such as neurons, glial cells, and neural stem cells) may lead to brain hypoplasia. IE2 is essential for lytic infection and viral latency reactivation, and it is also involved in HCMV development and host immune regulation [30]. In in vitro experiments, IE2-transduced NPCs produced fewer neurospheres and smaller neurospheres than control cells, demonstrating that IE2 inhibits self-renewal and growth of neural progenitors [20]. Based on the fact that HCMV has strict species specificity, there is currently no suitable animal model to study the specific in vivo mechanism of IE2-induced nervous system damage. However, in this study, we constructed transgenic mice (Rosa26-LSL-IE2^+/−^, Camk2α-Cre), and we found that long-term HCMV-IE2 expression inhibits the proliferation and differentiation of neural stem cells, stimulates the activation of microglia and astrocytes in the nervous system, produces an inflammatory response, further leads to the death of neurons, disrupts the normal development of neural tissue, and ultimately leads to the occurrence of microcephaly.

Microcephaly is a neurodevelopmental condition that is characterized by a severe reduction in postnatal brain size [31]. In our study, the brain tissue weight, whole brain size, and brain volume of IE2-expressing mice were smaller than those of the control mice. Moreover, the thickness of the cerebral cortex is significantly decreased in IE2-expressing mice. Although cortical lamellar, intermediate, and ependymal/subependymal areas exhibited different degrees of reduction from stage P2 through P10, the structure of each layer is relatively clear. However, it is unclear whether brain damage is directly caused by IE2 expression. Albeit, our previous study demonstrated that the presence of HCMV-IE2 can affect the animals’ spatial memory and learning ability in adult mice [22], which suggests that HCMV-IE2 damage to the nervous system persists. In addition, in another model that IE2 is continuously expressed in liver tissue, we first observed that all IE2-expressing mice died in the late embryonic stage, and secondly, the weight of the liver of the IE2-expressing mice was significantly reduced as compared with the control mice [21]. Based on these findings, we speculated that newborn microcephaly was produced by long-term steady expression of HCMV-IE2 in the brain.

The expansion and filling of neurons are essential to maintaining brain volume. Oh et al. [32] had confirmed neuronal loss to cause microcephaly research. In our study, we also found that the number of neurons at each time node was reduced in IE2-expressing mice compared with the control mice. The specific expression of Camk2α in neural stem cells and neurons [33] led to the activation of IE2 transcription and translation processes in both cells of our mouse model. Camk2α and IE2 co-staining results confirmed the expression of IE2 and Camk2α in neurons (Supplementary Figure 2). It is widely known that increased cell apoptosis and inhibited cell proliferation are two major causes of cell loss. Firstly, caspase-3 confirmed increased apoptosis in IE2-expressing mice. Furthermore, Ki67, SOX2, and PAX6 immunofluorescence staining indicated that proliferation of NPCs was impaired, suggesting that IE2 expression inhibits the proliferation of NPCs. In vivo studies in primates and rodents have demonstrated that CMV infection in the first trimester is mainly concentrated in the subventricular zone which is enriched with NSCs [34–36]. Consistent with our study, Dasol Han et al. [20] demonstrated that HCMV-IE2, but not IE1, impairs the in vitro proliferation of neural progenitor cells. Furthermore, we found that TBR1 were evidently increased in IE2-expressing cells, indicating that IE2 expression may block the differentiation of neurons, which is also a basis for neuronal loss.

Additionally, we also observed the damage to the neural stem cells and neurons in the cerebral cortex of IE2-expressing mice. Furthermore, we also found another interesting observation that the area of the hippocampus was smaller in IE2-expressing mice from stage P2. The subependymal area and the niche areas of neurons in the DG area of the hippocampus [37, 38], we analyzed from the neural stem cells, neurons, and cell proliferation, all showed that neurogenesis was significantly damaged (results not shown). This also confirms that sustained expression of HCMV-IE2 can remarkably impair neurogenesis.

The homeostasis of the central nervous system depends to a large extent on the balance of innate immunity. Astrocytes and microglia have singly greater role in supporting and nourishing neurons and protecting the homeostasis of the nervous system [4, 39, 40]. Once the homeostasis of the central nervous system is disrupted, microglia are activated first, and over-activated microglia release pro-inflammatory factors to damage neurons and activate astrocytes, and over-activated astrocytes further aggravate neuronal damage, which will further damage the nervous system [41]. Therefore, some researchers believe that the glial cells can release a large number of inflammatory factors, causing an imbalance of immune homeostasis in the brain, causing damage to neurons, interfering with the normal development of the brain, and further worsening the deterioration of microcephaly [42]. Lum et al. [43] demonstrated that the activation of microglia inevitably causes irreversible damage to the nervous system during the neurodevelopmental processes of young mice. In our study, we observed that in IE2-expressing mice, IBA-1-labeled microglia increased significantly, and the morphology also changed from branched to round as from stage P4. Meanwhile, CD68 is microglia M1 type marker, and its increased expression suggests that microglia are activated to a pro-inflammatory state. Furthermore, GFAP, a marker for reactive astrocytes, was significantly increased in IE2-expressing mice. Thus, we confirmed that IE2 expression disrupted neural homeostasis and further accelerate to brain damage.

Our study confirms the in vivo mechanism of HCMV-IE2-induced neonatal microcephaly and provides a pathological mechanism for the development of nervous system malformations caused by HCMV infection during pregnancy. In addition, the study includes a number of limitations. First, considering the size and complexity of the HCMV genome, our findings cannot adequately explain the mechanisms underlying the neurodevelopmental impairment of congenital HCMV infection. Second, due to lack of studies, after infection with HCMV, it is uncertain how much IE2 is expressed in people. The third, due to the limitations of the current transgenic technique, there were fewer transgenic positive mice. Consequently, the precise processes are unknown. Therefore, the findings of this study merely provide conjectures on how IE2 could affect the neurological system following HCMV infection. More clinical and epidemiological data are required to prove the impact of HCMV infection on pregnancy. In the following work, we will construct an in vivo time-expressed drug-induced stable expression of IE2 transgenic mouse model in vivo to better explain the clinical manifestations and specific pathogenic mechanisms of nerve damage caused by HCMV infection at different stages of pregnancy.

In conclusion, we successfully constructed transgenic mice (Rosa26-LSL-IE2^+/−^, Camk2α-Cre) possessing long-term and consistent expression HCMV-IE2 in the brain tissue. Our findings, based on this transgenic mouse model, show that long-term IE2 expression may cause neonatal microcephaly. The expression of IE2 induces the proliferation and differentiation of neural stem cells, resulting in the activation of microglia and astrocytes in the nervous system and resulting in the death of neurons and disruption in the normal development of neural tissue, and eventually leads to microcephaly. In addition, our findings provide pertinent molecular explanations for the developmental abnormalities of the newborn nervous system brought on by intrauterine HCMV infection during pregnancy, which advances our knowledge of the underlying cellular and molecular processes of HCMV infection.

## Supplementary Information

ESM 1

## Data Availability

The datasets generated during and/or analyzed during the current study are available from the corresponding author on reasonable request.
