# Supplementary material for: Human Cytomegalovirus IE2 Disrupts Neural Progenitor Development and Induces Microcephaly in Transgenic Mouse
Source: Mol Neurobiol. 2023 Mar 29;60(7):3883–97. doi: 10.1007/s12035-023-03310-1 (PMC10224843; doi:10.1007/s12035-023-03310-1)
Supplement: Supplementary file 1 — ESM 1 [file 12035_2023_3310_MOESM1_ESM.pdf]

Supplementary Figure

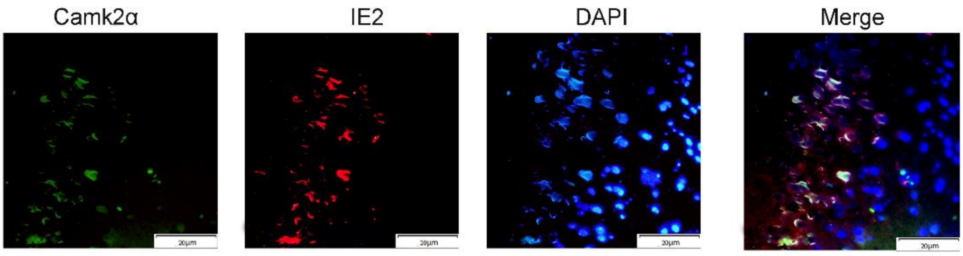

Supplementary Figure1 Images of the cerebral cortex was co-stained with Camk2α and IE2 at phases P2 in Rosa26-LSL-IE2+/-, Camk2α-Cre mice. Camk2α (greed).IE2 (red). DAPI (blue). Scale bar:20um

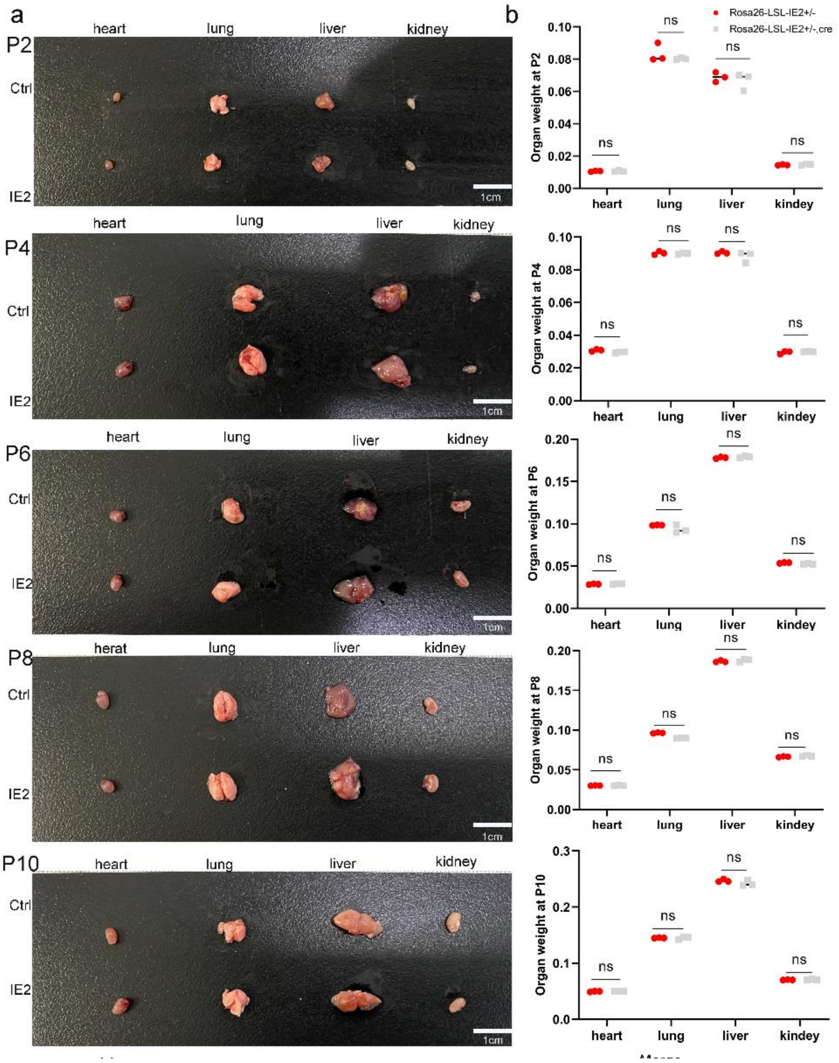

Supplementary Figure2 Other organs' weight and size are unaffected by HCMV-IE2.

(a) Morphology of different organs (heart, lung, liver, kidney) at stages P2, P4, P6, P8 and P10. (b) Weight of organs (heart, lung, liver, kidney) at stages P2, P4, P6, P8 and P10. n=3, the number of P2, P4, P6, P8, P10 different organs (heart, lung, liver, kidney). Scale bar:1cm. Ctrl represents Rosa26-LSL-IE2$^{+/-}$ mice, IE2 represents Rosa26-LSL-IE2$^{+/-}$, Camk2α-Cre mice.

ns: ns, no statistical difference

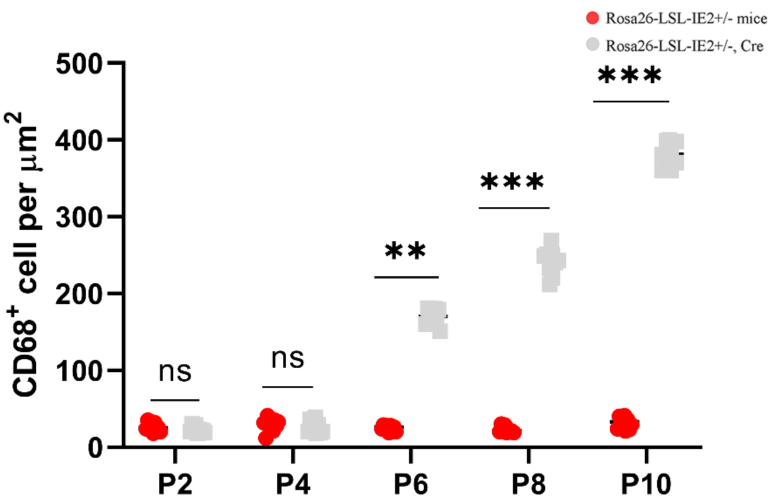

Supplementary Figure3 Measurement of CD68⁺ cells per μm^2 in the cerebral cortex

Measurement of CD68⁺ cells per μm^2 in the cerebral cortex at stages P2, P4, P6, P8, and P10 from the experiment presented in Figure 7b. P2: n=9/3; P4: n=9/3; P6: n=9/3; P8: n=9/3; P10: n=9/3. n: number of slices/different brains.
